# Protocol to quantify compound retention in *Drosophila melanogaster* for the pharmacokinetic study of orally administered compounds

**DOI:** 10.1016/j.xpro.2025.103974

**Published:** 2025-07-23

**Authors:** Nadiia Sadova, Bernhard Blank-Landeshammer, Julian Weghuber

**Affiliations:** 1 Center of Excellence Food Technology and Nutrition, University of Applied Sciences Upper Austria, Stelzhamerstrasse 23, Wels 4600, Austria; 2 FFoQSI GmbH-Austrian Competence Centre for Feed and Food Quality, Safety and Innovation, Technopark 1D, Tulln 3430, Austria

**Keywords:** Cell Biology, Metabolism, Model Organisms

## Abstract

*Drosophila melanogaster* is a valuable tool for intestinal function research. Here, we present a compound retention assay (CORE) to quantify the orally administered compounds that were absorbed, metabolized, or accumulated in the tissues (for example, fat) of *D. melanogaster*. We describe steps for preparing flies and media, compound administration, and sample collection. We then detail procedures for determining concentrations and calculating CORE values. This protocol circumvents the limitations of bioavailability research in the fruit fly, such as administering precise compound amounts.

For complete details on the use and execution of this protocol, please refer to Sadova et al.^1^

## Highlights

• Procedure for non-invasive compound retention quantitation in the fruit fly
• Instructions for compound administration to the fruit flies for pharmacokinetic studies
• Steps for the customization of compound solvents and extraction
• Protocol that enables multiple compound administration and analysis simultaneously

## Abstract (editor highlights)

Publisher’s note: Undertaking any experimental protocol requires adherence to local institutional guidelines for laboratory safety and ethics.

## Graphical abstract

**Figure:**
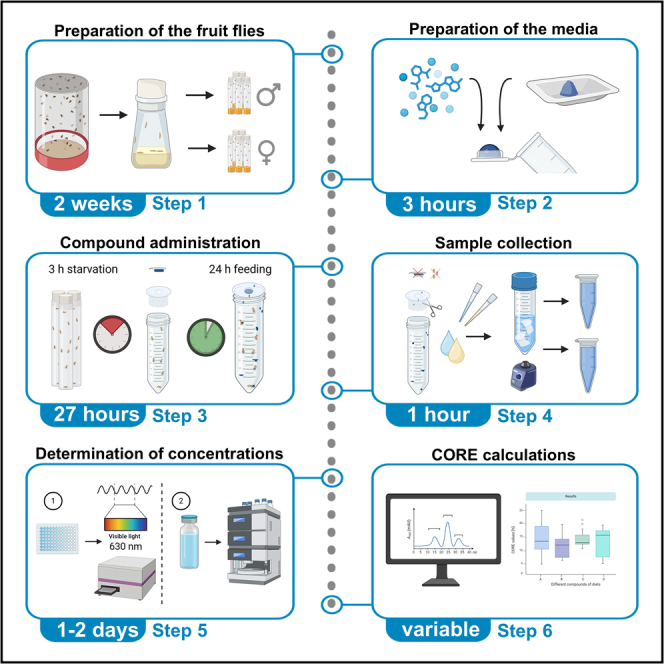

## Before you begin

*Drosophilamelanogaster* (*D. melanogaster*, the fruit fly) is a well-recognized model organism for intestinal function research, as well as for the investigation of various bioactive molecules, such as artificial drugs and naturally occurring bioactive molecules. The protocol below describes the specific steps to apply *D. melanogaster* for the quantitation of the absorption, accumulation or excretion of the bioactive compounds, which were orally administered to a fruit fly.

### Institutional permissions

This study, conducted with *D. melanogaster* in Austria, did not require national or institutional approvals, but researchers should obtain necessary approvals as per their local and institutional guidelines if required.

### Preparation of *Drosophila* cultivation media

**Timing: 1–2 days**

This step describes the preparation of the fruit fly media used for cultivating fruit flies and isolating age-synchronized fruit flies. The described media can be made in advance and stored sealed at 2°C–8°C for up to 3 months.

1. Weigh dry media ingredients as described in the section ‘materials and equipment’ in the labeled 1 l beakers. Choose the beakers with consideration that the prepared medium should take ca. ½ of the beaker volume.
2. Prepare larval cornmeal medium:
  a. Add water and stir the medium without heating until no clumps remain.
  b. Tightly cover the beaker with the aluminum foil and autoclave it at 105°C for 15 min.
    ***Note:*** We do not recommend using a heating plate for this type of medium because the high viscosity of the cornmeal makes stirring hard and causes medium burns at the bottom of the beaker.
  c. Allow the autoclaved media to cool off to 70°C with constant stirring.
  d. Add propionic acid and methylparaben solution and stir well.
  e. Homogenize medium using an immersion blender if necessary.
  f. Pour medium into stock bottle (∼25−50 ml/bottle).
  g. Cover with cheese clothes and let cool off and solidify until the next day.
  h. Store sealed and refrigerated at 2°C–8°C.
3. Prepare sugar-yeast medium:
  a. Add water and stir the medium without heating until no clumps remain.
  b. Tightly cover the beaker with the aluminum foil and bring it to a boil using a heating and stirring plate and a stirring magnet.
  c. Once agar foam starts climbing the beaker, remove the medium from the heat, remove the aluminum foil, and let it cool off to 70°C with constant stirring.
  d. Add propionic acid and methylparaben solution and stir well.
  e. Pour medium into the stock bottles (∼25−50 ml/bottle) and/or narrow vials (∼5 ml/vial).
  f. Cover with cheese clothes and let cool off and solidify until the next day.
  g. Store sealed and refrigerated at 2°C–8°C.
4. Prepare egg-collection grape agar medium:
  a. Add 500 ml of water to a 1 l beaker, stir, and add the powdered grape agar without heating until completely dissolved.
  b. Tightly cover the beaker with the aluminum foil and bring to a boil, take the beaker off the heat while stirring, and then bring to a boil for a second time, as required by the manufacturer (Genesee Scientific, USA).
  c. Cool media to ∼60°C while stirring (yet avoid formation of bubbles).
  d. Pour medium into sterile Petri dishes (∼6 ml per Petri dish). Let it cool and solidify, then cover with a petri dish lid.
  e. Store sealed upside down, refrigerated at 2°C–8°C.

***Note:*** Fruit fly cultivation media and egg collection agar can be replaced with comparable recipes that have already been established and normally used for standard fly culturing. However, if the media with different ingredients and caloric values are applied, it may change the duration of larval development. The timing in this protocol is optimized for the usage of the medium recipes described above.

### Preparation of the age-synchronized and sex-sorted fruit flies for the CORE experiments

**Timing: 14 days**

This step describes the preparation of the sex-sorted, age-synchronized fruit flies cultivated at 25°C, 60% relative humidity (RH), 12:12 h light-dark cycle.

5. Set up a large-scale culture to harvest synchronized F1 progeny:
  a. Mix 2 g of active dry yeast with 4 ml of water to achieve peanut butter consistency.
  b. Place a smudge of the yeast paste in the center of the grape agar Petri dish.
  c. Anesthetize 200–300 young male and female parental fruit flies.
  d. Transfer the fruit flies into the egg cage and top it with the yeast-agar plate.
  e. Place the egg cage on the side until all the flies are awake.
  f. Bring the egg cage into the climate chamber and place it vertically, so that the Petri dish is at the bottom. Leave for ∼24 h.
  g. Replace the agar plate next day with a fresh one without the yeast paste. Leave in the climate chamber for another 20–22 h.

***Note:*** Do not exceed 22 h at this step, as the eggs will start hatching after ∼24 h at +25°C.

6. Prepare a batch of fruit flies that will later be applied in the CORE experiment:
  a. Collect the eggs from the agar plate using a paintbrush cleaned with ethanol and sterile Dulbecco’s phosphate-buffered saline (DPBS) into 15 ml tube.
  b. Wash the eggs with DPBS until the supernatant becomes clear.
  c. Remove most liquid from the eggs and dispense 40 μl of eggs into each stock bottle with the larval medium.
    ***Note:*** For pipetting *Drosophila* eggs, use wide-opening pipette tips or cut off the first 2 cm of the 1 ml pipette tip. Pipetting yields the best results (homogenous firm egg mass without transparent fluid layers), when the pipette plunger is released in one sharp, quick move for both collecting eggs in the pipette tip and for releasing eggs onto the medium.
    ***Note:*** One such stock bottle will provide approximately 200–250 female and 150–200 male fruit flies of the strains like ORC or *w*^*1118*^. Adjust the number of stock bottles based on the desired experiment design and strain.
  d. Incubate the stock bottles in the climate chamber for 10 days.
7. Transfer one-day-old eclosed flies to the sugar-yeast medium in the stock bottles and allow them to mate for 2 days.
8. Sort age-synchronized flies into groups of 30 males or 30 females.
9. Place sex-sorted synchronized flies on the sugar-yeast medium in the narrow vials and cultivate them until the desired experimental age, e.g., 5–7 days.

***Note:*** If the experiment is conducted with the fruit flies older than 7 weeks, double the planned number of flies to compensate for the population’s mortality.

### Testing whether the compound of interest is consumed and is potentially toxic

**Timing: 1 week**

To ensure the success of the CORE experiments, the compound consumption must be confirmed. We recommend using the compounds’ concentrations that are not acutely toxic for the adult flies. If the compound with known toxic activity is intentionally tested to investigate its excretion, we advise proceeding with caution, as a damaged intestinal barrier can lead to false CORE results. The simple diagnostic for the intestinal barrier damage is done by means of identifying the Smurfs – the fruit flies that turned blue after the consumption of the nonabsorbable reference dye, as described by Rera and colleagues.^2^ This step of the protocol describes how the consumption of the medium with the tested compound can be confirmed, as well as helps identify the first signs of acute toxicity.

10. Prepare 4-5 vials with 30 male or female fruit flies of the same age as described in the previous section.
11. Prepare sugar-yeast medium enriched with the tested compound and the reference dye:
  a. Follow the steps 3a-d.
  b. At the temperature <70°C, add the compound of interest at the tested concentration(s) and 1% of reference dye.
  c. Stir well to completely and homogenously incorporate the test compound and the reference dye.
  d. Pour medium into the narrow vials (∼5 ml/vial).
  e. Cover with cheese clothes and let the medium cool off and solidify until the next day.
  f. To prepare the medium for the control group, follow steps 3a-d and add 1% of the reference dye at a temperature <70°C. If the tested compound is provided as an aqueous or ethanolic solution, substitute it in the control group medium with an applied solvent, for example, water or ethanol.
  g. Store sealed refrigerated at 2°C–8°C.
12. Test media consumption and toxicity of the administered compound:
  a. Transfer the experimental flies to the prepared media and rear them for 1 week in standard conditions.
  b. Transfer the flies to the fresh media every 2-3 days.
  c. Write down the number of the dead flies in each vial. At the end of the week, calculate the percentage of dead and alive flies in each treatment group and compare them using a fitting statistical test, e. g. t-test or one-way ANOVA.
  d. Write down the number of the fruit flies that turned blue (Smurf). At the end of the week, calculate the percentage of Smurf flies in each treatment group and compare them using a fitting statistical test, e.g. t-test or one-way ANOVA.
    ***Note:*** The presence of over 10% of the flies in the treatment group exhibiting the Smurf phenotype suggests that the compound compromises the intestinal barrier integrity. Rupture of the intestinal barrier is a sign that a tested compound is not suitable for the CORE experiments.
  e. Observe whether there are blue excreta dots on the vial plugs and walls after the flies were allowed to eat for 24–48 hours and whether the dark blue gut line is visible in the abdominal area of the fruit flies. This indicates that the dyed medium with the tested compound was consumed.

## Key resources table

**Table:** 

REAGENT or RESOURCE	SOURCE	IDENTIFIER
**Chemicals, peptides, and recombinant proteins**		

Brilliant blue for coloring food (FCF) (reference dye)	Carl Roth	2981.4
D(+)-Saccharose, ≥ 99.5%	Carl Roth	4621.2
D(+)-glucose, anhydrous	Carl Roth	X997.2
Agar-agar, I kobe	Carl Roth	5210.4
4-Hydroxybenzoic acid methyl ester, ≥ 98%	Carl Roth	3646.1
Propionic acid, ≥ 99.5%	Carl Roth	6026.2
Ethanol 99.9% HPLC grade	Riedel-de Haen	34963–2.5
Inactive dry yeast	Genesee Scientific	62–106
Yellow cornmeal	Genesee Scientific	62–100
Active dry yeast “Red Star”	Genesee Scientific	62–103
Nutri-Fly grape agar	Genesee Scientific	47–102
DPBS	PAN-Biotech	P04-36500

**Experimental models: Organisms/strains**		

*D. melanogaster*, e.g., strain Oregon-R-C, males and females	Bloomington Drosophila Stock Center	BDSC: 5; FlyBase: FBsn0000277

**Other**		

Plate reader POLARstar Omega	BMG Labtech	736–0374
Climate chamber HPP750 eco	Memmert	HPP750
LDPE flange cap with internal diameter 25.1 mm, flange diameter 31.8 mm, height 16.8 mm	MOC product company (Mocap)	FCS1.000NA1
Steel hole puncher for plastics with 6 mm diameter nozzle	Amazon	N/A
Narrow *Drosophila* vials, 30 ml	Nerbe Plus	11-881-0021
*Drosophila* stock bottles, 170 ml	VWR	734–1249
Embryo collection cage, small, for 60 mm Petri dish	Genesee Scientific	59–100
Flugs stock bottle plugs, 37 mm	Genesee Scientific	49–100
Flugs narrow plastic vial plugs, 25 mm	Genesee Scientific	49–102
Cotton vial plugs	Genesee Scientific	51–101
Synthetic paintbrush	Amazon	N/A
Dissecting needle 140 mm	VWR	233–0102
Screw cap 50 ml tubes	Greiner	227261
Screw cap 15 ml tubes	Greiner	188271
Sterile tubes 1.5 ml	Greiner	616201
Sterile tubes 0.5 ml	Greiner	667201
Petri dishes, 60 mm	Greiner	628102
96-well transparent flat-bottom plates	Greiner	655101

## Materials and equipment

**Table:** *Drosophila* larval medium composition

Reagent	Final concentration	Amount
Saccharose	3%	15 g
Inactive yeast	2.5%	12.5 g
Agar-agar	1%	5 g
Yellow cornmeal	6%	30 g
Glucose	5.5%	27.5 g
Propionic acid	0.48%	2.4 ml
10% 4-hydroxybenzoic acid methyl ester in ethanol	1%	5 ml
Tap water	N/A	500 ml
**Total**	N/A	**∼500 ml**

Store at 2°C–8°C for up to 3 months.

***Note:*** Total volume considers the loss of the evaporated water after boiling and cooling off the medium.

***Alternatives:*** All *Drosophila* food ingredients can be replaced with identical ingredients from the available manufacturers.

**Table:** *Drosophila* sugar-yeast medium composition

Reagent	Final concentration	Amount
Saccharose	5%	25 g
Inactive yeast	10%	50 g
Agar-agar	1.5%	7.5 g
Propionic acid	0.48%	2.4 ml
10% 4-hydroxybenzoic acid methyl ester in ethanol	1%	5 ml
Tap water	N/A	500 ml
**Total**	N/A	**∼500 ml**

Store at 2°C–8°C for up to 3 months.

***Note:*** Total volume considers the loss of the evaporated water after boiling and cooling off the medium.

***Alternatives:*** All *Drosophila* food ingredients can be replaced with identical ingredients from the available manufacturers.

**Table:** *Drosophila* medium composition for toxicity and consumption tests

Reagent	Final concentration	Amount
Saccharose	5%	25 g
Inactive yeast	10%	50 g
Agar-agar	1.5%	7.5 g
Propionic acid	0.48%	2.4 ml
10% 4-hydroxybenzoic acid methyl ester in ethanol	1%	5 ml
Brilliant blue FCF	1%	5 g
Test compound	“X”%	$\frac{500\;ml}{100\%}\times X\%$
Tap water	N/A	500 ml
**Total**	N/A	**∼500 ml**

Store at 2°C–8°C for up to 3 months.

**CRITICAL:** Avoid confusion of Brilliant Blue FCF (FCF stands for “Fit for Coloring Food”) reference dye, also known as Blue 1 or E133 (C.I. 42090) and Coomassie Brilliant Blue R-250 or G-250 (C.I. 42660 and C.I. 42655). This protocol employs Brilliant Blue FCF (further “reference dye”), which is also applicable in the coloring of food. It is non-toxic for the fruit flies and is not absorbed by healthy guts.

***Note:*** Total volume considers the loss of the evaporated water after boiling and cooling off the medium.

***Alternatives:*** All *Drosophila* food ingredients can be replaced with identical ingredients from the available manufacturers.

**Table:** 10% (weight per volume) 4-hydroxybenzoic acid methyl ester in ethanol

Reagent	Final concentration	Amount
4-hydroxybenzoic acid methyl ester (methylparaben)	10%	100 g
Ethanol 99.9%	N/A	1,000 ml
**Total**	N/A	**1,000 ml**

Store at 20°C–25°C for up to 1 year.

**Table:** Brilliant blue FCF stock solution, 100 mg·ml^−1^

Reagent	Final concentration	Amount
Brilliant blue FCF (reference dye)	**100 mg·ml**^**-1**^	10 g
dH_2_O	N/A	100 ml
**Total**	N/A	**100 ml**

Store at 2°C–8°C for up to 6 months.

**CRITICAL:** This solution is used for the calibration curve for the absorbance quantification; therefore, the weight and volume must be measured with maximum possible precision.

## Step-by-step method details

### Preparation of experimental *Drosophila* media

**Timing: 3 h**

This step handles the preparation of the *Drosophila* media necessary to conduct the CORE experiments. The media described in this section do not contain sensible amounts of proteins, fats, or dietary fiber present in whole yeasts, and consist of water, sugar, and agar, which create a matrix for the tested compound and the reference dye. This medium should be freshly prepared to avoid drying and shrinking.

1. Prepare the medium ingredients suggested in Table 1.

**Table 1:** Example of *Drosophila* experimental medium composition for powdered or fluid tested compounds

Reagent	Concentration	Examples of media	
		0.1% tested compound (administered as powder)	0.1% tested compound (administered as 10% aqueous solution)
Saccharose	5%	5 g	5 g
Agar-agar	1.5%	1.5 g	1.5 g
Propionic acid	0.48%	0.48 ml	0.48 ml
10% 4-hydroxybenzoic acid methyl ester in ethanol	1%	1 ml	1 ml
Brilliant blue FCF (reference dye)	0.2%	0.2 g	0.2 g
Tested compound	X%	0.1 g	1 ml
dH_2_O	N/A	98.52 ml	97.52 ml
**Total**	N/A	**∼100 ml**	**∼100 ml**

Store at 2°C–8°C for max. 1 week.

***Note:*** The concentrations of the tested compound can be adjusted to the specific goals. If multiple concentrations of the tested compound in the solution or liquid extract are compared, it is advisable to subtract the amount of the applied solvents from the amount of water in each medium.

2. Before cooking the medium, prepare the medium carriers:
  a. Take 20–30 of 0.5 ml tubes for each tested compound.
  b. Place them in the tube stand open so that the medium can be pipetted into the cap.
3. Prepare medium matrix:
  a. Weigh agar and saccharose in the dry 50 or 100 ml beaker.
  b. Add deionized water.
  c. Add a stirring magnet and stir well.
  d. Cover the medium and bring it to a boil.
  e. Cool off the medium to 70°C or slightly below.
  f. Add propionic acid and 4-hydroxybenzoic acid methyl ester ethanolic solution.
  g. Keep the medium on the stirring plate until step 5.
4. Prepare the compound of interest:
  a. If the tested compound is a powder, weigh and note the exact amount of compound of interest.
    **CRITICAL:** It is critical to obtain the precise mass of the added compound. For that, weigh the weighing paper before and after the compound was added, as well as when the compound was transferred to the medium, to estimate the amount of compound remaining on the weighing paper.
    ***Note:*** A lot of dry powders are electrostatic and hold onto weighing paper, tray or surrounding surfaces. For best results, use an anti-static gun or brush.
  b. If the administered compound is a solution with an exact known concentration, move to the next step.
5. Prepare the reference dye for each medium:
  a. Weigh the exact amount of the reference dye that needs to be added to the medium.
    **CRITICAL:** It is critical to obtain the precise mass of the added compound.
    ***Note:*** Brilliant Blue FCF used as a reference dye is electrostatic and easily spreads on the gloves, weighing table, and holds onto weighing paper or tray. For best results, use an anti-static gun or brush.
  b. Write down the exact masses of the empty weighing tray and the tray with the reference dye.
6. Weigh the exact amounts of the medium (100% - percentage of the tested compound mass - percentage of the reference dye mass) for each tested compound or concentration in the 50 ml beakers.
7. Add reference dye and compound of interest to each beaker and vigorously stir.

**CRITICAL:** It is essential to homogeneously distribute the reference dye and the compound of interest. Ensure, they do not adhere to the walls of the beaker.

8. While the medium is still fluid, pipette it into the caps of the prepared 0.5 ml tubes.

***Note:*** For best results, cut off the tip of the 1 ml pipette tip or the tips with a wide opening.

**Figure 1:**
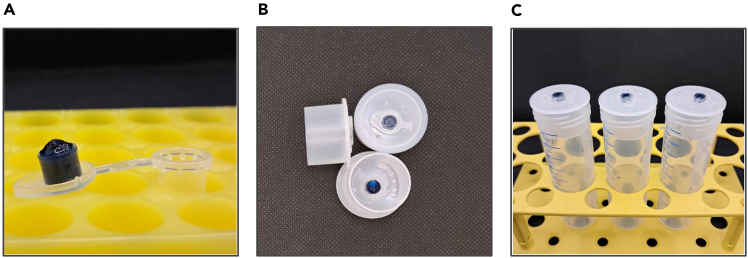
Detailed demonstration of the CORE assay setting (A) Photo of the liquid dyed medium applied to a tube cap before solidifying. (B) The flanged cap with the experimental medium insert. (C) A complete experimental setting with the fruit flies and medium insert.

***Note:*** As the medium cools off, it shrinks. For this reason, it is recommended to pipette above the level of the tube cap (see Figure 1A). However, care should be taken not to spill the medium outside the cap.

9. Let the medium cool off and solidify for at least 1 h before the CORE experiments.

### Compound administration to the fruit flies

**Timing: 3 h + 24 h**

This major step allows the evaluation of the absorption-excretion pharmacokinetics of the orally administered compounds in the fruit fly. The compound retention values (CORE) reflect compound bioavailability in the matrix of the described medium. Different food matrices (composition, texture, presence of fiber or fat), as well as other factors (such as temperature in the climate chamber, age of the flies, their microbiota), can change the bioavailability of some compounds. For example, fat-soluble vitamins can be better bioavailable if the medium contains fat, which can also be investigated using the CORE assay.

10. Starve the fruit flies prepared for the experiment for 3 h:
  a. Transfer 7-day-old fruit flies of the same sex in groups of 30 to the empty 50 ml tubes at 10:00 am
  b. Cover with a breathable cotton plug.
    **CRITICAL:** The flanged caps by MOC (see key resources table) are compatible with most 50 ml conical centrifuge tubes. Before the experiment begins, ensure that the flanged caps and the tubes have compatible diameters, and the tube can be centrifuged.
    ***Note:*** The starting times suggested in this step are best suited to perform and complete the entire experiment during the workday times (7 a.m. to 4–5 p.m. of the next day).
  c. Save screw caps from the 50 ml tubes for the later steps.
  d. Place starving flies in the climate chamber for 3 h until exactly 1:00 pm.
11. Prepare flanged caps with experimental media prior to the experiment (see Figure 1B):
  a. Perforate each flanged cap in the middle with a steel hole puncher using a 6 mm nozzle.
  b. Pierce 5-6 small holes on the top of the flanged caps with a dissecting needle for air circulation.
  c. Disinfect the caps with 70% ethanol and paper towels.
  d. Cut off the caps of the 0.5 ml tubes with experimental media with scissors.
  e. Carefully place them inside the punched holes in the flanged caps.

**CRITICAL:** The dyed medium can stain surfaces it contacts. Take great care not to touch the flanged cap with the medium and not to smear some of the dye on the cap.

12. Introduce the medium to the prepared flies:
  a. Replace the cotton plug with the flanged cap and a medium insert (see Figure 1C). For that, gently tap the 50 ml tube against the mouse pad as during the standard vial flipping.
  b. Place the fruit flies back in the climate chamber for 24 h, allowing them to eat and excrete. Refer to troubleshooting 1, if after 24 h, there are no traces of food consumption.

***Note:*** This protocol is not designed to quantify the consumed food, nor to control the feeding behavior in the fruit flies. The feeding behavior of the flies remains a variable that depends on many factors, such as diet, health status of the flies, the tested compound of interest, etc. However, using our protocol, one can compare the feeding behaviors of the fruit flies between the groups, if the different diets or supplemented compounds are included in the study, by using the proportion of the reference dye mass to the entire feed mass or ratios of the reference dye between the groups. We recommend applying food quantification of solid media with caution, or referring only to the consumed dry substance. The reason for this is the water evaporation from the agar-based medium over time. If the supplemented compound alters the feeding behavior of the flies, e.g., reduces appetite, the reporting of such changes in future studies is advisable.

### Sample collection

**Timing: 1 h**

13. Inspect the fruit flies for the Smurf phenotype.
14. Terminate the experiment in the replicates with Smurfs and refer to troubleshooting 2.
15. After 24 h, discard the experimental flies or transfer them to the standard sugar-yeast medium in case of repeated measurements / longitudinal studies.

***Note:*** If the solid medium frequently falls out of the insert during these manipulations, refer to troubleshooting 3.

16. Cut the flanged cap in half with the scissors or the lancet, avoiding contact with the excretion spots as much as possible.
17. Clean the scissors between the replicates with a paper towel if necessary.
18. Place cut flanged cap halves inside the respective 50 ml tubes and top the tube with the screw cap from step 9b.
19. Add the appropriate sample solvent to the tube and tightly close the screw cap.
  a. Vigorously vortex tubes to completely dissolve blue excreta spots.
  b. Centrifuge the tubes for 5 min at 4,100× g to collect solution drops on the bottom of the tube.
  c. Collect 2 aliquots of the transparent supernatant for the next steps.

***Note:*** The collection procedure has to be evaluated for each target compound and depends on its solubility and applied concentration. For water-soluble compounds of interest, choose dH_2_O as a solvent. For compounds with intermediate polarity, the addition of polar organic solvents (e.g., 70% ethanol in dH_2_O) might be necessary. Lipophilic compounds of interest are best collected using a liquid-liquid extraction procedure, employing a binary or ternary system of immiscible solvents.^3^ In these cases, target compounds are retained from the organic phase, while the reference dye is collected from the aqueous phase. For the solvent suggestion, see Table 2.

***Note:*** The volume of the solvent has to be established individually, dependent on the initial concentration of the compound of interest in *Drosophila’s* medium, however, we do not recommend a volume smaller than 1 ml to ensure optimal sample collection. In the initial study^1^ we have applied 1.5 ml dH_2_O per tube. The more solvent is used, the lower the concentration of the excreted target compound will be. This aspect has to be fitted to the selected follow-up analytics (HPLC-MS, GC-MS, etc.) and their sensitivity.

***Note:*** When a liquid-liquid extraction procedure was chosen, typically two phases will form: one aqueous phase containing the hydrophilic reference dye and one organic phase containing the lipophilic compound of interest. Therefore, aliquots of each phase must be taken in order to quantify the reference dye and the compound of interest.

**Table 2:** Sample solvent suggestions for the CORE assay in the fruit fly

Collection procedure	Suggested solvents	Chemical characteristics of the fitting compounds	Compound examples
Single-phase extraction	Deionized water	Readily water-soluble, neutral compounds	Sugars, alcohols, glycosylated polyphenols, etc.
	30–70% ethanol, methanol or acetonitrile in deionized water	Neutral compounds with intermediate to low polarity	Flavonoids, polyphenol aglycones, isoprenoids
	Deionized water + 0.1% acetic acid	Basic compounds with high to intermediate polarity	Basic amines and alkaloids such as morphine, atropine, and scopolamine
	Deionized water + 0.1% ammonia	Acidic compounds with high to intermediate polarity	Fatty acids, terpenes, glucuronidated polyphenols, acidic alkaloids such as theophylline
Biphasic extraction	Methyl-tert-butyl ether, methanol, water (10/3/2.5, v/v/v)^4^	Lipophilic compounds	Acylglycerols, phospholipids, ceramides, fat-soluble vitamins
	Chloroform, methanol, water (2/2/1.8, v/v/v)^5^		

### Quantification of the reference dye and compound of interest concentrations in the excreta samples

**Timing: 2 h + variable**

20. Prepare solutions for the calibration curve of reference dye:
  a. Use a prepared stock solution to make a 100 μg ml^−1^ solution.
  b. Make 450 μl of reference dye solutions with concentrations 0, 2, 4, 6, 8, and 10 μg ml^−1^ if measured in duplicates.
  c. Add 200 μl of volume for each further technical replicate (e. g. 650 μl if measured in triplicates).

***Note:*** Consider the composition of the solvent applied for the sample collection. The standard solutions for the calibration curve have to be prepared in the same solvent to have the same background absorbance.

***Note:*** To avoid evaporation of the solvent, always prepare the calibration solutions fresh before the measurement.

21. Define the optimal sample dilution:
  a. For the first time measurement, measure the absorbance of a few undiluted, as well as diluted 1:2, 1:4 and 1:8 sample to define the optimal sample dilution.
  b. Pipette 200 μl of calibration solutions and diluted samples in technical duplicates into the transparent 96-well plate.
  c. Include a solvent blank as a concentration point 0.
  d. Measure the absorbance at 630 nm using a plate reader.

***Note:*** The aim is to select the dilution where the majority of the samples have absorbance within the detection range, closer to the center of the calibration line. We found sample dilution 1:4 to be optimal for excrete samples, which were collected with 1.5 ml of solvent (excrete of 30 flies for 24 h).

22. Quantify the reference dye concentration in the samples:
  a. Dilute all the samples to the selected degree using the same solvent as the one used for the preparation of the calibration solutions.
  b. Pipette 200 μl of calibration solutions and diluted samples in technical duplicates to the transparent 96-well plate.
  c. Include a clear solvent blank as a concentration point 0.
  d. Measure absorbance at 630 nm using a plate reader.
  e. Construct the calibration line plotting dye concentrations on the X-axis and absorbance values on the Y-axis.
  f. Interpolate the unknown concentrations of the samples using absorbance values.
  g. If samples were diluted, multiply the obtained concentration by the dilution factor.
23. Tailor the analytical approach of compound quantification to the chemical parameters of the selected compound and available analytical equipment.

**Figure 2:**
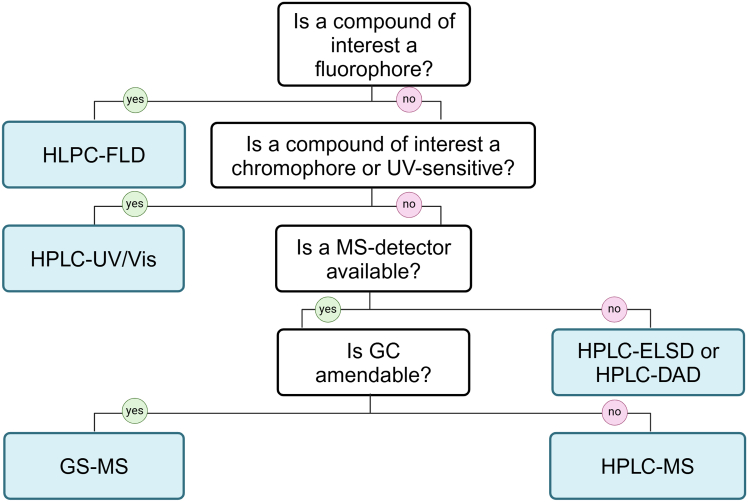
Basic decision tree on the analytical technique for target compound(s)

***Note:*** In our experience, for natural organic compounds and phytochemicals, separation either by HPLC or GC is necessary to ensure adequate selectivity and sensitivity. In some cases (e.g. compounds with strong fluorescent properties or pigments in high concentrations) sole usage of a photometer or a plate reader may be sufficient. For a basic guide to deciding which detection technique to use, see Figure 2. If no compound of interest was detected in the excrete samples refer to troubleshooting 4.

### Calculation of the CORE values

**Timing: variable**

24. Convert obtained gravimetric dye and compound masses in the solid medium to the amount of substance using the respective molar mass, e.g. 1 g of the reference dye in the food refers to $\frac{1\;g}{792.84\;\frac{g}{mol}}\;\approx 1.261\;mmol$.
25. Express the measured concentrations of the excreted dye and compound of interest in the units with identical volume unit (e.g. μg ml^−1^ and mg ml^−1^).
26. Convert the obtained excreted dye and compound masses to the amount of substance using respective molar mass.
27. Calculate the CORE value using the formula below. If the obtained CORE value is negative, refer to troubleshooting 5.
  $$CORE\left[\%\right]=100\%-\frac{n_{excreted\;compound}\times n_{reference\;dye\;in\;medium}}{n_{compound\;in\;medium}\times n_{excreted\;dye}}\times 100\%\text{,}$$
  where $n_{excreted\;compound}$ is the amount of the excreted substance, $n_{reference\;dye\;in\;medium}$ is the amount of the dye in the solid medium, $n_{compound\;in\;medium}$ is the amount of the compound of interest in the solid medium, $n_{excreted\;dye\;}$ is the amount of the excreted dye.
  ***Optional:*** For multiple chemical forms of the administered compound (e. g. aglycone and glycoside of the same compound) added to the *Drosophila’s* medium:
  a. Sum amounts of the substance of the chemical forms of the administered compound in *Drosophila’s* medium.
  b. Sum amounts of the substance of the chemical forms of the compound in the *Drosophila’s* excrete.
  c. Calculate CORE values with the following equation:

$$CORE\left[\%\right]=100\%-\frac{\left(n_{excreted\;aglycone}+n_{excreted\;glycoside}\right)\times n_{reference\;dye\;in\;medium}}{\left(n_{aglycone\;in\;medium}+n_{glycoside\;in\;medium}\right)\times n_{excreted\;dye}}\times 100\%\text{,}$$

where $n_{excreted\;aglycone},n_{excreted\;glycoside}$ are the amounts of the excreted compounds of interest, $n_{reference\;dye\;in\;medium}$ is the amount of the dye in the solid medium, $n_{aglycone\;in\;medium},n_{glycoside\;in\;medium}$ are the amounts of the compounds of interest in the solid medium, $n_{excreted\;dye\;}$ is the amount of the excreted dye.

***Note:*** For the detailed mathematical derivation of the provided formulas, refer to Sadova et al.^1^

## Expected outcomes

**Figure 3:**
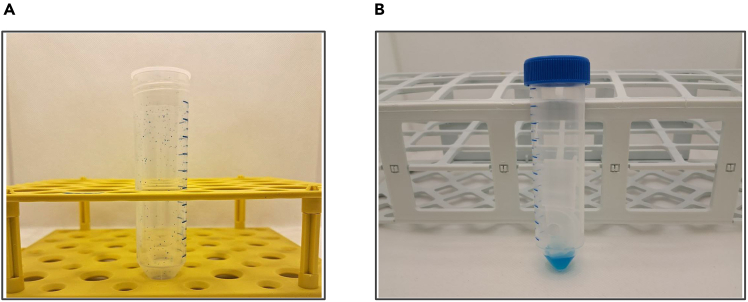
Examples of visual outcomes of a CORE experiment (A) Characteristic blue excreta with the reference dye confirming the consumption of the medium. (B) Complete collection of the excreted metabolites and dye.

Successful performance of the CORE experiment is characterized by multiple excreta spots containing the reference dye on the walls of the collection tube (Figure 3A) and by complete collection of the dyed sample from the tube (Figure 3B). The CORE value is a fraction of the whole and, therefore, expected to range from 0 to 100%. This value depends on the age, sex, dietary matrix, and intestinal health status of the fruit flies. For this reason, it is not an absolute compound parameter, but a relative one.

## Limitations

This protocol is best suitable for the compounds of interest that can be homogenously incorporated in the water-based *Drosophila* medium. Therefore, the application of the fat-soluble compounds, compounds in the paste-like, highly viscous consistency (e.g. pure lutein oil) is limited for this protocol and needs to be individually tested by analyzing the compound concentration in the different media areas within one batch. Due to the recommended light-dark incubation of the flies, the CORE assay is limited to the compounds that are light-stable for at least 48 hours. The applied reference dye is not absorbed and is excreted only by the specimens with an intact intestinal barrier. If during the CORE experiment, the Smurf phenotype is observed (reference dye has leaked through the ruptured barrier into the hemolymph of the fruit fly), the experiment has to be terminated as the results will not be valid.

## Troubleshooting

### Problem 1: Poor consumption of the experimental medium

Poor medium consumption can be related to the gustatory characteristics of the administered compound (bitter taste, repelling aroma) and the health status of the experimental flies.

### Potential solution

**Figure 4:**
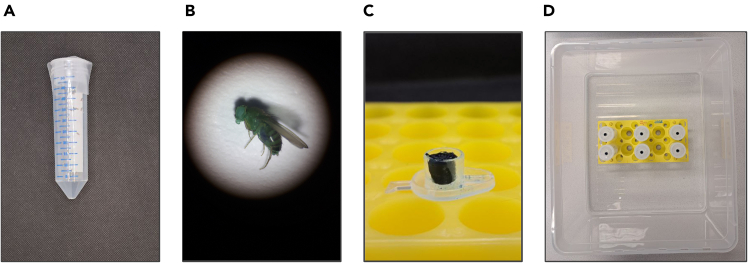
Illustration of selected troubleshooting issues and solutions (A) Experimental setting for the weakened or old *D. melanogaster*. (B) Example of the Smurf fruit fly. (C) Extreme shrinkage of the experimental medium due to dry air. (D) Additional humidity is added via the tray with water.

• Repeat the consumption confirmation step, if necessary, where flies are introduced to the dyed food in the conditions they are used to, e.g. food is on the bottom of the regular fly vial. Observe if the dyed excreta will appear on the vial walls and plug surface after ∼24 h.
• If the weak, sick, or old flies are used, it might be challenging for them to climb up the tube to the food source. To overcome this problem, use adhesive tape to firmly attach the flanged cap to the 50 ml tube without blocking the air holes, and afterward place the 50 ml tube horizontally to ease food access (Figure 4A).

### Problem 2: The experiment is regularly terminated because of observed Smurfs

The fruit flies with the leaky intestinal barrier (Figure 4B) are unsuitable for the CORE experiments and are the reason to terminate the experiment or exclude the biological replicate. While it is not uncommon to have 1-2 young Smurfs in the population of a few hundred flies, it is a reason for concern if multiple replicates are regularly discarded for this reason.

### Potential solution

• If a mutant strain is applied in the CORE experiment, ensure that the strain is not known for a particularly weak intestinal barrier (reduced intestinal stem cell proliferation, septate junction knockouts, etc.).
• If the problem occurs during the application of the robust wild-type strain, consider that the tested compound, even if not acutely toxic, may cause intestinal barrier damage. To confirm it, test the experimental food without the compound of interest vs. the food with the compound for ∼1 week and score Smurf flies daily. In our experience,^6^^,^^7^ normal population aged 5–7 days will have <4% of Smurfs at 25°C.

### Problem 3: The experimental medium falls out of the tube cap

The experimental medium is meant to be attached to the top of the vial, allowing flies to excrete below it. One of the possible complications of the CORE experiment is the agar medium falling out of the cap during the feeding phase or further manipulations. It can be explained by inadequate pipetting temperature or inadequate storage conditions that promote rapid medium dehydration and shrinkage (Figure 4C).

### Potential solution

• Pipette liquid experimental medium at temperatures under 68–70°C, when the medium is viscous.
• Always prepare media fresh and do not store them longer than 1 week. During one week of storage, make sure the 0.5 ml tubes are tightly closed and additionally sealed in the zip-lock bag. Store media in the fridge.
• If the medium shrinkage is traced back to the 24 h of incubation time, control that the fly climate chamber indeed supports at least 60% of relative humidity. If the fruit flies are kept in the lab without a climate chamber, try to place the tube rack on a tray filled with water (Figure 4D).

### Problem 4: No compound found in the excreta

When no compound was found in the excreta, the formal logical assumption is that the bioavailability of the compound was 100%, and all of it was retained by the fruit fly. However, it is rarely the case. It is likely that the administered concentration is too low to be detected after excretion.

### Potential solution

• Try reducing the volume of the sample solvent to 1 ml or 0.5 ml.
• If the administered compound is not toxic and available in the big amounts, increase the concentration of the compound in the food.
• If the first two options are not possible, increase the number of flies in each group from 30 to 50.
• If that does not solve the problem, increase the experiment duration. Consider changing the medium caps every 24 h to prevent the shrunken medium from falling out. Combine this approach with reduced sample solvent to increase the concentration of the excreted compound. E.g., in the initial experiment (24 h) 1.5 ml of solvent per sample was used. In the next experiment setting, try 48 hours and use 1 ml of solvent for each tube.

### Problem 5: The CORE value is a negative number

In experiments, where a complex multi-component drug is added to the fly food, it is possible to observe a negative CORE value if the experimental medium contains the substrate for the compound of interest. For example, in the publication by Sadova et al.,^1^ we fed the flies with soy extract that contained both a glycosidic form of isoflavones and their aglycone. Glycosides were deglycosylated by the intestinal microbiota and partly absorbed or excreted. This way, when measured, it appeared that the flies had excreted more aglycones than they had consumed.

### Potential solution

If more than one pure compound is being tested, first, run a chemical analysis on the composition of the administered drug or extract. Should the analysis reveal the presence of multiple chemical forms of the same compound (glycosides and aglycones), it may be meaningful to evaluate them both together. Otherwise, microbial deglycosylation or any other kind of microbial biotransformation within the intestine can lead to false results. Consider confirming the results in the germ-free version of the applied strain or incubating the fly gut microbiota with the tested compounds.^1^

## Resource availability

### Lead contact

Further information and requests should be directed to the lead contact, Julian Weghuber (julian.weghuber@fh-wels.at).

### Technical contact

Technical questions on executing this protocol should be directed to and will be answered by the technical contact, Nadiia Sadova (nadiia.sadova@fh-wels.at).

### Materials availability

This study did not generate new unique reagents.

### Data and code availability

This study did not generate new datasets/code.

## Acknowledgments

This research was funded by Christian Doppler Research Association (Josef Ressel Centre for Phytogenic Drug Research, Wels, Austria, and Josef Ressel Centre for Innovation in Bioavailability Research, Wels, Austria). The financial support by the Austrian Federal Ministry of Economy, Energy and Tourism, the National Foundation for Research, Technology and Development, and the Christian Doppler Research Association is gratefully acknowledged. In addition, this work was supported by the Austrian Competence Centre for Feed and Food Quality, Safety and Innovation (FFoQSI). The COMET-K1 Competence Centre FFoQSI is funded by the Austrian ministries BMVIT, BMDW and the Austrian provinces Lower Austria, Upper Austria, and Vienna within the scope of COMET Competence Centers for Excellent Technologies. The program COMET is handled by the Austrian Research Promotion Agency FFG. The graphical abstract and figures were created and edited using BioRender.com (2025).

## Author contributions

N.S.: conceptualization, methodology, visualization, writing – original draft, and writing – review and editing; B.B.-L.: supervision and writing – review and editing; J.W.: funding acquisition, supervision, and writing – review and editing.

## Declaration of interests

The authors declare no competing interests.
